# Treatment of Osteochondral Fracture of the Lateral Femoral Condyle with TWINFIX Ti Suture Anchor “X”‐Shaped Internal Fixation under Arthroscopy: A Surgical Technique and Three Cases Report

**DOI:** 10.1111/os.12632

**Published:** 2020-03-11

**Authors:** Song Zhou, Min Cai, Ke Huang

**Affiliations:** ^1^ Investigation performed at Center for Joint Surgery Third Affiliated Hospital of Guangxi Medical University, Nanning Second People's Hospital Nanning China

**Keywords:** Arthroscopic, Internal fixation, Osteochondral fracture, Suture anchor, TWINFIX Ti

## Abstract

Osteochondral fracture of the lateral femoral condyle is a rare intra‐articular injury with or without patellar dislocation. This kind of disease is commonly seen in the knee joint sprain during strenuous activity. At present, open reduction is often used to treat osteochondral fractures. In recent years, with the development of arthroscopy, we have been able to complete the reduction and internal fixation of fractures under arthroscopy. In this paper, three cases of osteochondral fracture of lateral femoral condyle were treated with arthroscopic TWINFIX Ti suture anchor internal fixation, and good results were obtained. After operation, the fracture of femoral condyle healed well and the function of knee joint recovered gradually. Suture anchor system is mostly used to repair rotator cuff and patellar tendon. This is the first case to apply the suture anchor system to the reduction and fixation of fracture.

## Introduction

Osteochondral fracture of the lateral femoral condyle is a rare injury of the knee joint, which mostly occurs in adolescence^1^. In adolescence, the cartilage‐bone interface is the weakest transitional area in the knee joint, and there is no obvious boundary between calcified and uncalcified cartilage^2^. The biomechanical strength of immature osteochondral junction was lower than that of mature osteochondral junction. Therefore, when shear stress is applied to the surface of the lateral condyle of the femur, its reaction is to break at the bone‐cartilage interface, rather than transfer the force to the bone substance^3^. This is also an independent anatomical factor for the vulnerability of bone and cartilage in this age group^4^. Although osteochondral fractures of lateral condyle of femur are rare, such diseases can cause significant knee joint pain and joint degeneration. With the improvement of medical processes, diseases in the knee joint are more and more inclined to be treated with minimally invasive treatment under arthroscopy.

## Case Report

**Figure 1:**
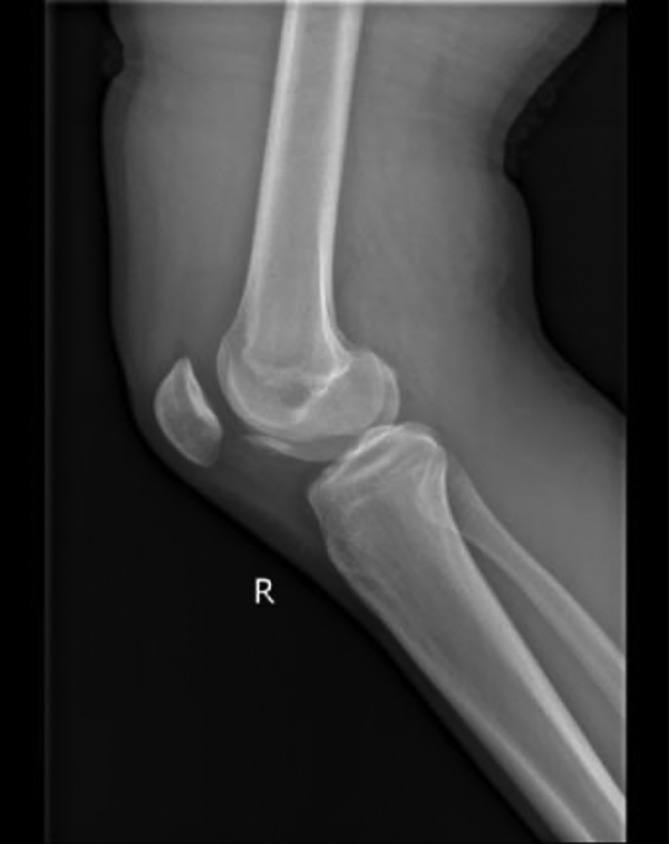
X‐ray examination of right knee joint: free bone mass can be seen at the anterior edge of the femur in the knee joint.

**Figure 2:**
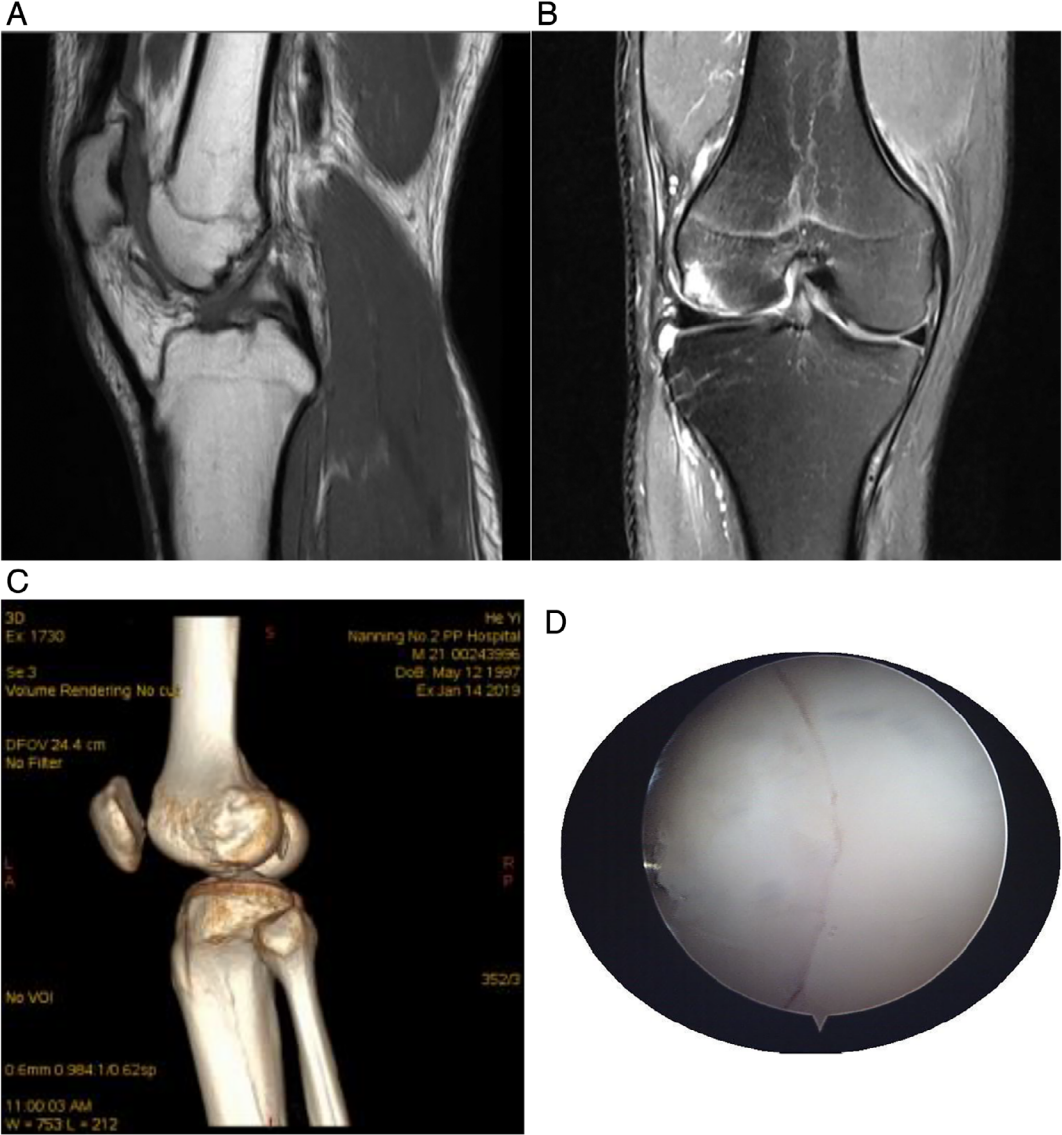
(A) MRI examination of the right knee joint: the bone continuity at the edge of the lateral condyle of the right femur was poor, the patchy high signal intensity was seen in the bone marrow cavity of the lateral condyle of the femur, and the local cartilage became thinner in the corresponding area. (B) MRI examination of the right knee joint: a patchy bone signal was seen in the anterior and lower part of the distal femur. (C) CT examination of the left knee joint: the continuity of the subarticular bone of the lateral condyle of the left femur was interrupted. (D) Under knee arthroscopy, obvious fracture line of lateral condyle of bone and osteochondral fracture of the lateral femoral condyle can be seen.

Case 1 The patient, an 18‐year‐old male, sprained his right knee when he jumped from a height of 2 m 2 days ago, causing severe pain in his right knee joint. Physical examination: swelling of the right knee, local tenderness, positive floating patellar test of the right knee, negative lateral stress test, negative rotation and extrusion test, and limited flexion and extension of the right knee joint. ROM (range of motion), F/E 90°/0°. The HSS (hospital for special surgery) knee score of knee joint was 58. X‐ray examination of the right knee joint showed poor bone continuity at the edge of the lateral condyle of the right femur and free bone fragment in the knee joint (Fig. 1). MRI examination: osteochondral fracture of right lateral femoral condyle and patellar contusion; right lateral collateral ligament injury; the injury of the anterior and posterior angle of the lateral meniscus of the right knee joint (grade II) (Figs 2A, 2B).

Case 2 The patient, a 21‐year‐old male, was injured in his left knee while playing football 10 days ago. After the injury, he felt pain in the left knee and his knee activity was slightly restricted. Physical examination: swelling of the left knee, local tenderness, and limited flexion and extension of the left knee joint. ROM, F/E 90°/0°. The HSS score of knee joint was 64. Computed tomography (CT) examination showed osteochondral fracture of the lateral femoral condyle (Fig. 2C).

Case 3 The patient, a 15‐year‐old female, sprained her left knee while playing badminton 3 days ago. These (case 2 and 3) patients have similar clinical symptoms and physical signs. ROM, F/E 100°/0°. The HSS score of knee joint was 67. Osteochondral fracture of the lateral femoral condyle of the left knee was found by knee arthroscopy (Fig. 2D).

## Surgical Treatment

**Figure 3:**
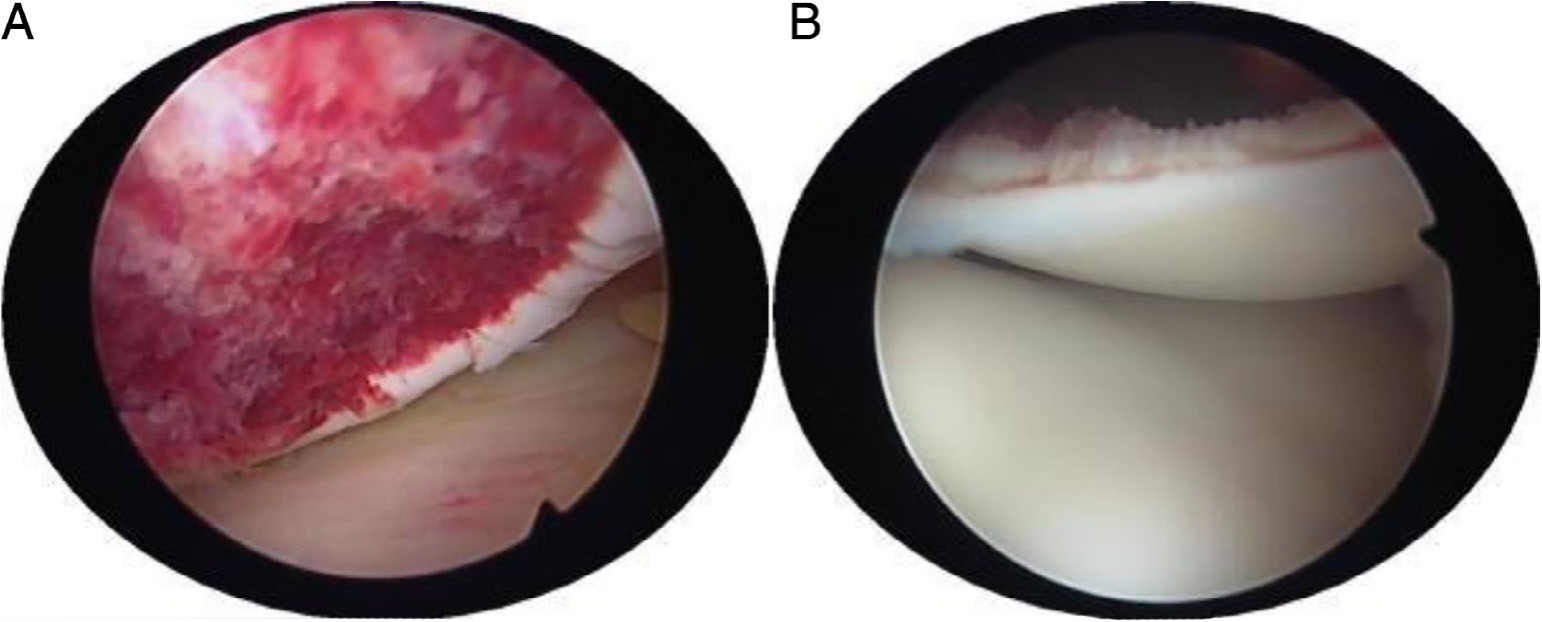
(A) The fresh 1.5 cm × 1.5 cm fracture surface of the lateral condyle of femur was found under arthroscopy. The bone mass is missing at the fracture. (B) 1.5 cm × 1.5 cm free bone was found in the knee joint cavity, and the bone fracture was intact.

The anterior medial and lateral approach of the conventional arthroscope was used to explore the location and size of the articular osteochondral injury and to find the free bone fragment (Fig. 3A,B). Knee joint placed on the edge of the operating table, natural prolapse. The flexion knee joint is convenient to expose the wound of the femoral condyle.

**Figure 4:**
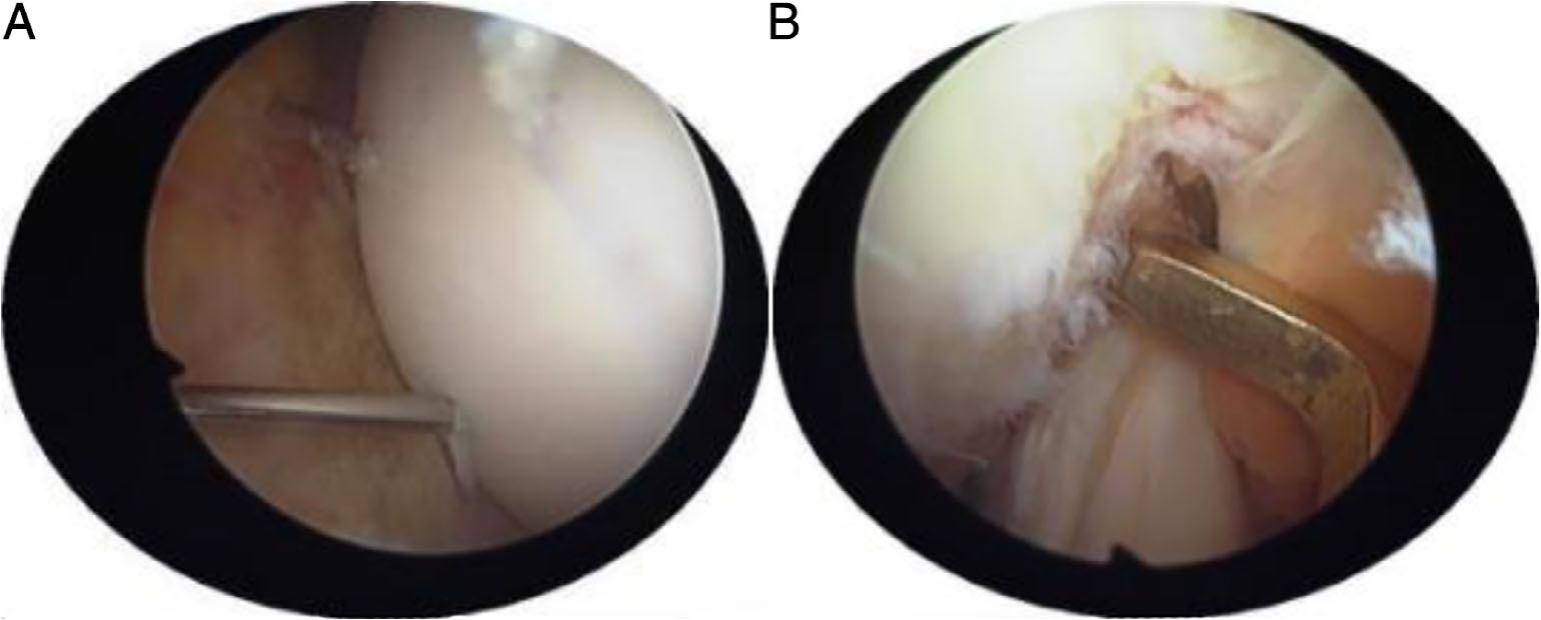
(A) One 1.5 mm Kirschner wire temporarily fixed the fracture block of the lateral condyle of the femur. (B) AIMER was located at the outlet of the medial bone canal of the lateral condyle of the femur.

During the arthroscopy procedure, the cartilage wound was repaired and cleaned, and the free bone fragment was reduced. After bone fragment reduction, to prevent bone fragment rotation, one or two 1.5 mm Kirschner wires were temporarily fixed in the fracture block of the lateral condyle of the femur (Fig. 4A). Then, according to the size of the fracture block, 3.5 mm (or 2.8 mm) wire anchor (TWINFIX Ti anchor; Smith & Nephew; Massachusetts; USA) was selected. After the nail entry point was selected, the anchor point was gradually enlarged with 1.0, 1.5, 2.0, and 2.5 mm Kirschner wires to avoid bone fragmentation. Through the operation of incision, the free bone fragment was fixed *in situ* to the lateral condyle of the femur with the help of arthroscopy. The tail of the anchor nail should be slightly lower than that of the cartilage surface.

**Figure 5:**
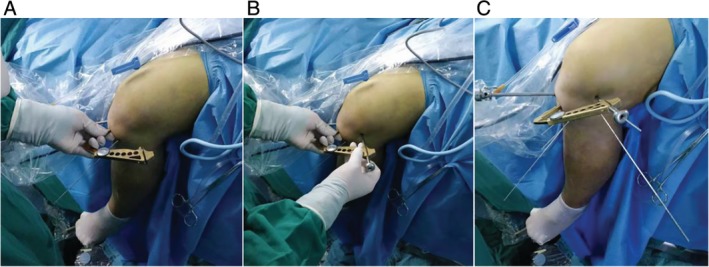
(A) Use of anterior cruciate ligament locator to assist drilling at the distal end of the femur. AIMER was located at the outlet of the medial bone canal of the lateral condyle of the femur, and the HANDLE was adjusted to a suitable angle (50°–60°). (B) BULLET fixed at the entrance of the lateral bone canal of the distal femur. (C) Making a small incision on the outside of the knee joint, it is convenient to drill two 2.0 mm bone channels from the distal end of the femur from the outside to the intercondylar fossa.

Determining the position of the medial exit of the two bone channels on the medial side of the lateral condyle of the femur, the anterior cruciate ligament locator was used to assist drilling (Fig. 4B). AIMER was located at the outlet of the medial bone canal of the lateral condyle of the femur, and the HANDLE was adjusted to a suitable angle (50°–60°) (Fig. 5A). BULLET was fixed at the entrance of the lateral bone canal of the distal femur (Fig. 5B). Making a small incision on the outside of the knee joint, it is convenient to drill two 2.0 mm bone channels from the distal end of the femur from the outside to the intercondylar fossa (Fig. 5C). The distance between the two bone canals should not be too small, generally around 1.5–2 cm. Exit the drill needle and insert a 2.0 mm hollow needle into the bone canal as a temporary working channel (can be replaced by an anesthetic puncture needle).

**Figure 6:**
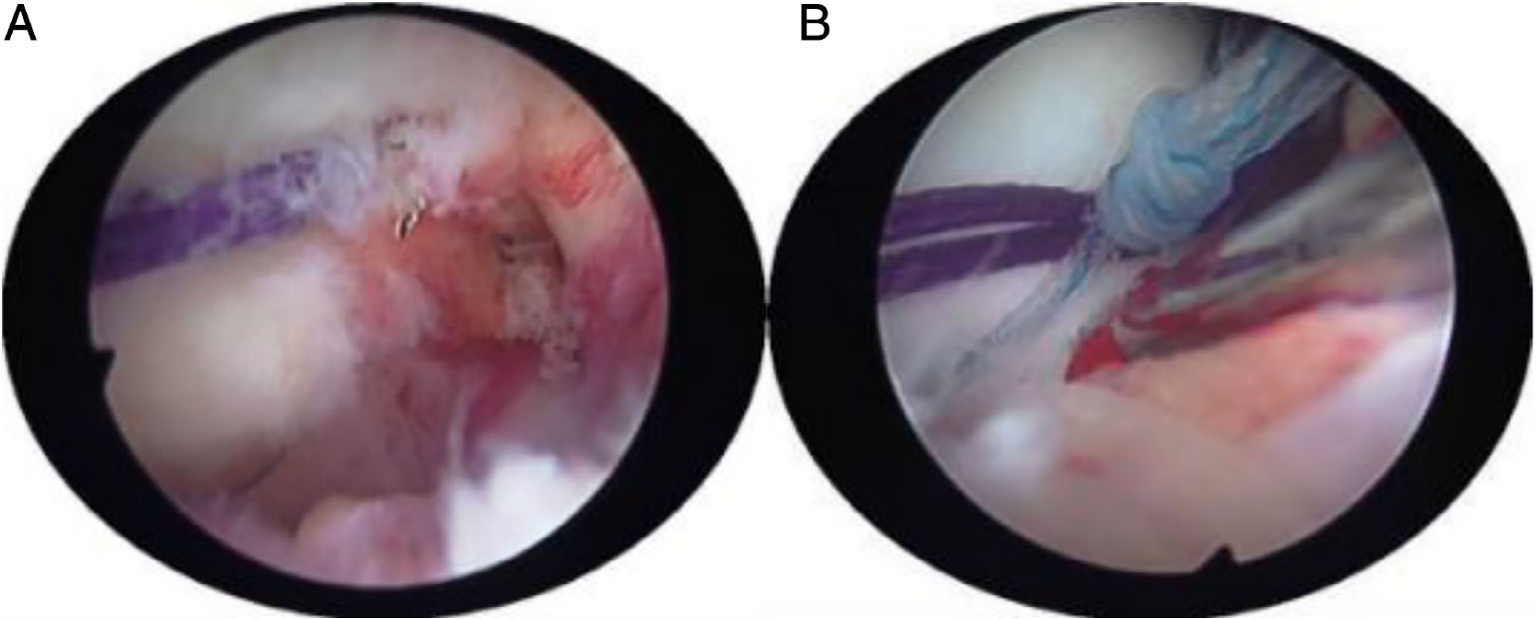
(A) Through the hollow needle channel of the femoral intercondylar fossa, the folding corner of the PDS line are exposed to the knee joint cavity through the bone canal. (B) The sutures passing through the bone canal and the corresponding sutures on the anchor were tightened and fixed.

First, fold the PDS (polydloxanone suture) line in half. Then, through the hollow needle channel of the femoral intercondylar fossa, the folding corner of the PDS line is exposed to the knee joint cavity through the bone canal (Fig. 6A). The folded PDS line enters the joint to form a coil (note that the end of the PDS line should be left outside the entrance of the femoral canal). By operating the incision, grab one of the sutures of the TWINFIX Ti suture anchor with a gripper, and then pass the suture through the coil of the PDS line. Finally, the PDS line is used as the guide line to bring the suture into the bone canal and outside the bone canal.

**Figure 7:**
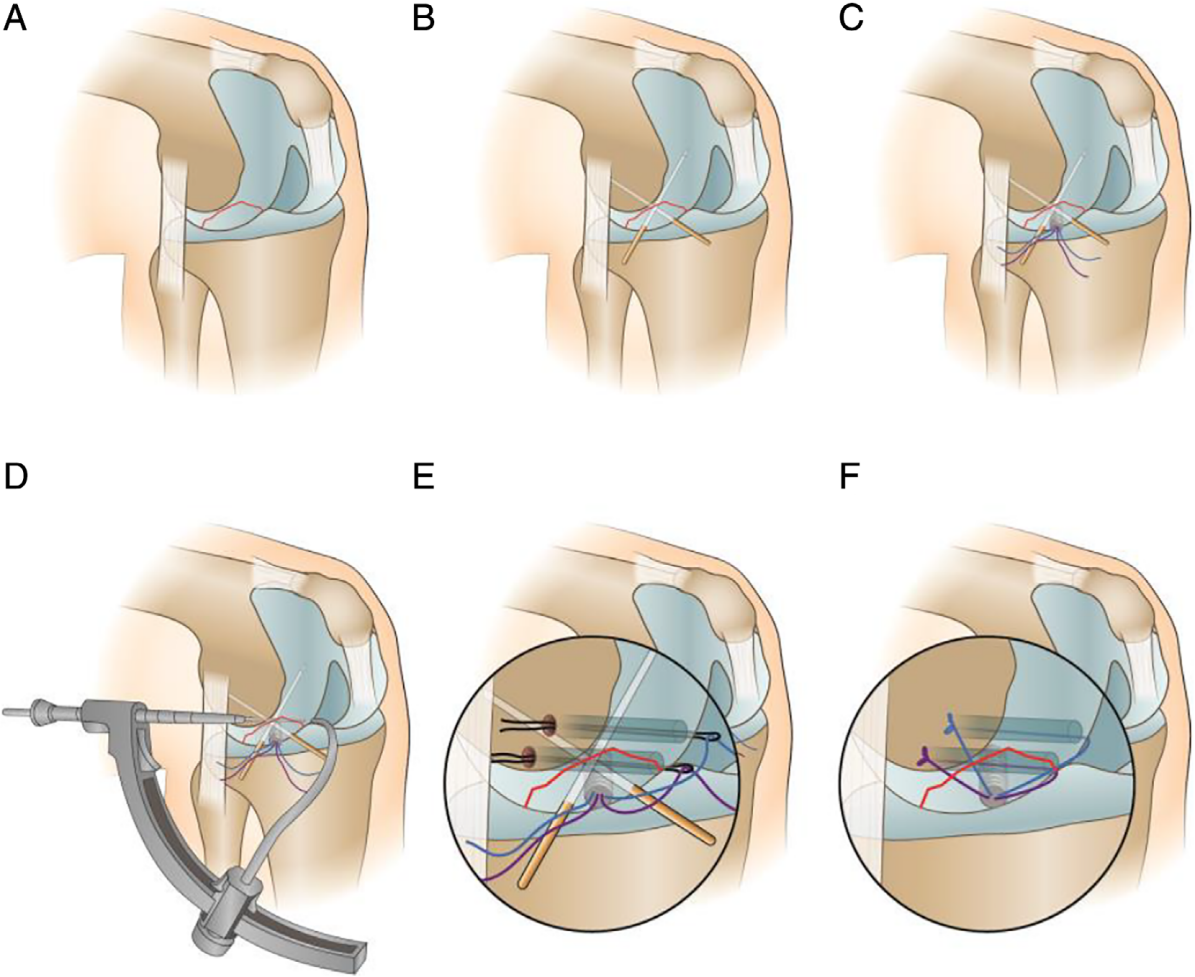
Surgical diagrams (A: osteochondral fracture of the lateral femoral condyle; B: fixation of fracture block with Kirschner wire; C: fixation of fracture block with anchor; D: preparation of bone tunnel; E: penetration of PDS line and PDS guidance of anchor suture to the outer entrance of femoral tunnel; F: Operation completion diagram).

The sutures passing through the bone canal and the corresponding sutures on the anchor were tightened and fixed in the lateral femur (Fig. 6B). Another suture is fixed in the same way. Remove the temporary fixed Kirschner needle. Check that the fracture is in good alignment and has solid fixation and smooth joint surface. The surgical diagrams are shown in Figs 7A–F.

## Outcome and Follow‐up

**Figure 8:**
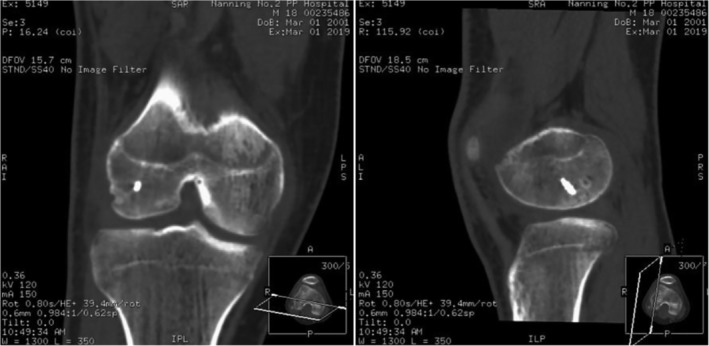
CT examination 6 months after operation: one screw internal fixation, regular external condyle cortex, good alignment at the end of fracture, callus growth and unclear fracture line could be seen in the right lateral femoral condyle.

Within 1 week after operation, the patients mainly underwent knee flexion and extension exercise in their hospital beds, and the range of motion of knee joint was between 10° and 90°. When the patients were reexamined 3 months after operation, the knee wore an adjustable brace for rehabilitation exercise and the patients could walk normally. The range of motion of the patients' knee joints are as follows (case 1‐3): F/E，0°‐130°/0°; 0°‐120°/0°; 0°‐130°/‐5°‐0°. There were no discomfort in the knee joints in the patients' daily activities. The knee joint HHS scores of the patients were as follows (case 1‐3): 93; 87; 92. The case 1 patient, 6 months later and CT examination showed that the cortex of the lateral condyle of the right femur was regular, the alignment of the fracture end was good (Fig. 8). Six months after surgery, patients have been able to carry out normal physical exercise, including playing basketball.

**Figure 9:**
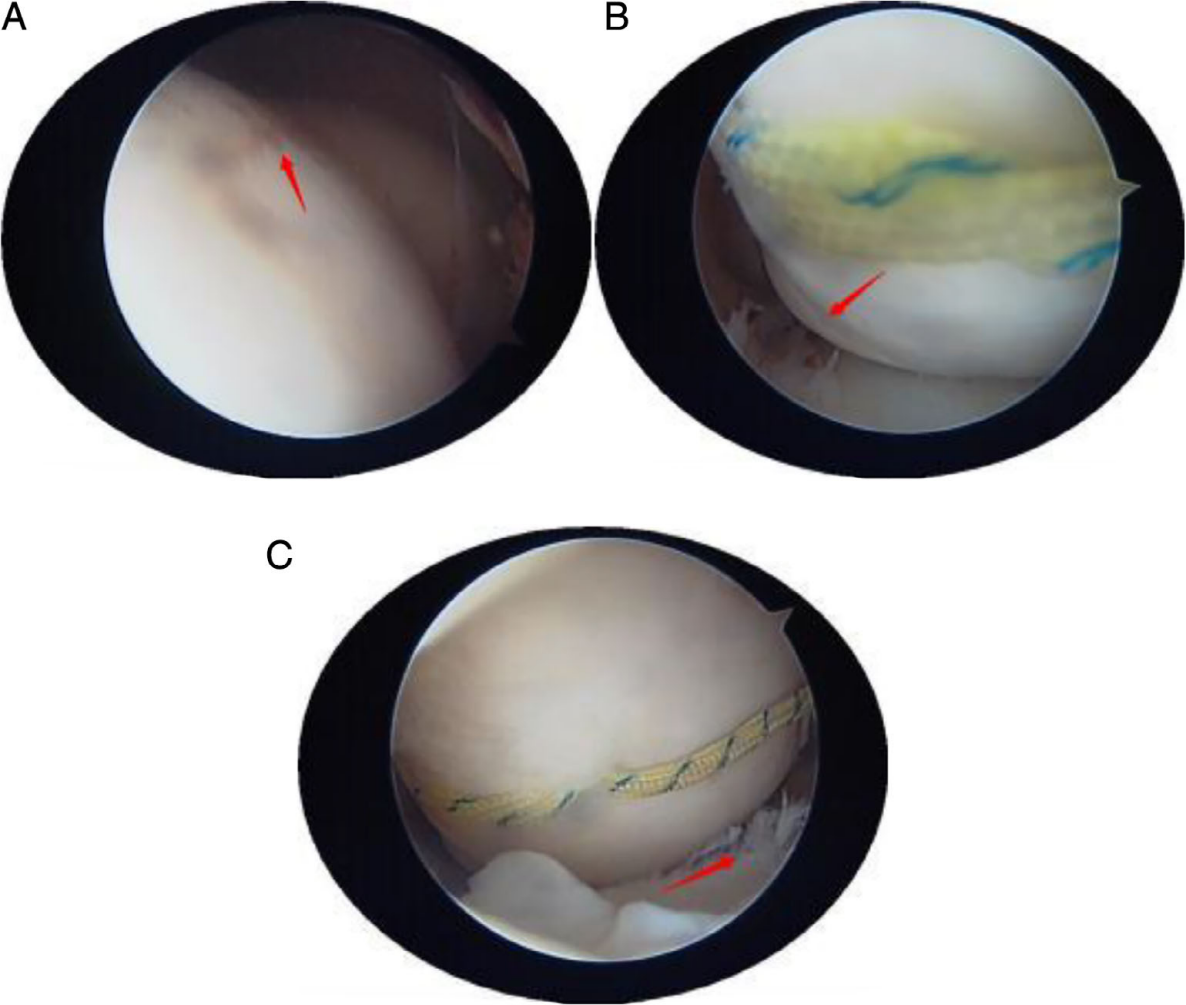
(A) A blurred fracture line can be seen at the fracture of the lateral condyle of the femur. (B) The suture of the lateral condyle of the femur is still fixed on the surface. (C) The free edge of the anterior foot of the meniscus was rough (degree I).

Now, 12 months after operation, in order to ensure the success of our operation and eliminate the adverse effects of anchor sutures on the knee joint, we invite patients to return to the hospital. We performed a second arthroscopic surgery on them with the consent of the patients. During the operation, we can see that the anchors and sutures are well fixed, and the fractures have healed, leaving only vague fracture lines. The outlet of the suture of the femoral surfaces were filled with soft tissue and the articular surfaces were flat (Fig. 9A, B). But in the case 2 patient, the free edge of the anterior foot of the meniscus was rough (degree I), and the body and posterior angle of the meniscus were not abnormal (Fig. 9C). There was no obvious abnormality in the articular surface of the tibia. The patient (case 2) said he returned to work early after the operation and began physical activity at 6 months. The injury of the anterior horn of the meniscus may be related to the early participation in physical activity and the suture fixed on the surface of the femur.

## Discussion

Intra‐articular osteochondral fracture has a certain ability for self‐repair. After acute articular surface fracture, the defect of bone and cartilage is filled with fibrin clot^5^. The defect area of the cartilage surface is in excess of 4 to 16 mm^2^, which is usually not the result of self‐repair^6^. For this kind of disease, the removal of free bone fragments is mainly used in. However, this method causes friction between the defective bone surface and tibia, which can easily lead to early osteoarthritis, especially the fracture of the load‐bearing surface of the knee joint. Therefore, only when the fracture block <1 cm^7^, or when the osteochondral fragments cannot be repaired, simple removal of the fracture block may be the choice of treatment^8^.

After comprehensive consideration, we decided to use the suture anchor system which adapts to the minimally invasive operation under the arthroscopy. The TWINFIX Ti anchor (Smith & Nephew) adopts high and low double helix structure. Therefore, the area of compressed bone around TWINFIX Ti anchor is larger and the anti‐pulling force is stronger. In the anchor cycle test under physiological load, the failure cycle of TWINFIX Ti anchor can reach 331 ± 190 cycles, which is good biomechanical stability compared with the general threaded anchor^9^. The anchor has no tail cap. The tail of the anchor screw below the surface of the cartilage of the femur can still provide a good fixation effect. Therefore, the suture anchor system does not have the problem that the tail of the nail does not match the surface of the joint cartilage. In addition, the Ultrabraid suture is very thin, even in the weight‐bearing area of the joint surface, and will not affect the leveling of the joint surface. Sutures in four different directions disperse the pressure caused by fracture fixation and reduce the cutting of cartilage.

In this case, we used a TWINFIX Ti suture anchor system anchor combined with double bone canal suture fixation of osteochondral fragments (Fig. 7). First of all, the anchor provides the initial fixation of a “point”, while the “X” cross fixation of double sutures and double bone channels forms a mesh “surface” with multi‐point comprehensive fixation. This surgical technique has not been reported in the previous literature.

The second knee arthroscopy proved that it was feasible to use TWINFIX Ti suture anchor “X” internal fixation in the treatment of osteochondral fracture of the lateral femoral condyle. The fixed sutures on the femoral surface did not have much effect on the joints, and the patients did not feel any discomfort. For such patients, we recommend that strenuous activity be avoided for 6 months after operation. In order to eliminate the effect of the suture on the articular surface and meniscus, the suture can be removed during the second arthroscopy within 6 months from the time of the operation.

### *Conclusion*

Suture anchor system has the advantages of less trauma, reliable fixation, no need to remove anchor, reduced complications, and so on. In this case, the knee joint function of patients recovered well after operation, and they could carry out daily life and physical exercise. The second arthroscopic operation found that the bone of the fractures healed well. Suture anchor system is feasible for the treatment of osteochondral fracture of the lateral condyle of femur.
